# Scope evolution at the journal of biological chemistry: Advancing molecular insight in a changing scientific landscape

**DOI:** 10.1016/j.jbc.2026.111427

**Published:** 2026-04-24

**Authors:** Henrik G. Dohlman, Alex Toker

For more than a century, the *Journal of Biological Chemistry* has served as a home for rigorous, mechanistic studies that define our understanding of biological processes. This commitment to molecular and biochemical insight remains the foundation of the journal.

What is changing is not that commitment, but how such insight is generated.

The molecular life sciences are undergoing a period of rapid transformation. Advances in computation, structural biology, large-scale experimental approaches, and integrative analyses are reshaping how we ask and answer biological questions. Increasingly, important discoveries emerge not from complete mechanistic dissection in a single study but from work that provides conceptual advances, establishes new frameworks, or generates hypotheses that guide subsequent investigation. This evolution presents both an opportunity and a responsibility. As a journal with a longstanding commitment to rigor and scientific leadership, JBC must ensure that it continues to capture the most meaningful advances in molecular and biochemical biology, while maintaining the standards that define its identity.

It is in this context that we are expanding the scope of JBC.

## A broader view of molecular insight

Historically, JBC has emphasized detailed mechanistic understanding as a central criterion for publication. That emphasis has served the field well and remains a defining strength of the journal. However, the pathways to mechanistic insight have diversified. In many areas of modern biology, particularly those involving complex systems, large datasets, or computational modeling, meaningful advances may precede full mechanistic resolution. Prominent examples include the human genome sequencing project, the development of new imaging methods like cryo-EM, and the creation of structure prediction tools like AlphaFold. These studies can reshape how we think about a biological process, identify key regulatory nodes, or provide the conceptual foundation upon which a mechanism is ultimately built. Recognizing this, JBC will more explicitly welcome studies that deliver conceptual advances, provide a foundation for mechanistic insight, employ rigorous approaches, and contribute enabling methods or resources. This is not a departure from our mission, but a refinement of how we interpret it.

## What is not changing

It is essential to be clear that expanding scope does not mean lowering standards. JBC will continue to prioritize rigorous experimental design and analysis, clear alignment between data and conclusions, transparency and reproducibility, and a focus on molecular and biochemical insight. Work that is purely descriptive, correlative without biological grounding, or incremental in nature will remain outside the scope of the journal. The defining characteristic of JBC is not any particular methodology, but the quality and depth of the insight it provides.

## Aligning with a changing field

This evolution reflects broader changes in the scientific ecosystem. The increasing scale and complexity of biological data, the integration of computational and experimental approaches, and the growing importance of translational and disease-relevant biology all point toward a more interconnected view of molecular science. At the same time, the publishing landscape is shifting. Authors are seeking venues that recognize diverse forms of scientific contribution, while maintaining rigorous standards of evaluation. For JBC, this means embracing a broader range of approaches while preserving the principles that have guided the journal for decades.

## The role of editors and reviewers

As JBC evolves, the role of our editors and reviewers becomes even more critical. Associate Editors will continue to serve as scientific gatekeepers, applying judgment to determine which studies meet the journal’s standards and align with its evolving scope. Editorial Board Members, in turn, play a central role in evaluating the rigor, validity, and significance of submitted work. Together, this community ensures that JBC maintains consistency, fairness, and scientific integrity across a wide range of disciplines.

## Looking forward

JBC has a long history of adapting to changes in the scientific landscape while remaining grounded in its core principles. The tools and approaches used to study biology will continue to evolve, but the need for careful, quantitative, and mechanistically informed understanding will not. By expanding our scope in a deliberate and disciplined manner, we aim to ensure that JBC remains a trusted venue for rigorous molecular and biochemical research, a home for emerging approaches that advance biological insight, and a leader in defining standards for scientific quality and reproducibility. This is an evolution, not a reinvention. It reflects our commitment to serving the scientific community by recognizing and publishing the most meaningful advances in modern biology.

We look forward to working with our authors, reviewers, and readers as we take this next step.

